# Dietary reprogram of macrophages for cell competition: a promising strategy for malignant cancer control

**DOI:** 10.1038/s41392-023-01664-5

**Published:** 2023-10-27

**Authors:** Jian Chen, Zhaoliang Su, Guangchuan Wang

**Affiliations:** 1 Department of Nephrology, Affiliated Hospital of Jiangsu University, Zhenjiang, 212013 China; 2 International Genome Center, Jiangsu University, Zhenjiang, 212013 China; 3 State Key Laboratory of Molecular Biology, Shanghai Institute of Biochemistry and Cell Biology, Center for Excellence in Molecular Cell Science, Chinese Academy of Sciences, Shanghai, 20031 China

**Keywords:** Cancer, Tumour immunology

In a recent study published in *Nature*, Zhang et al. explored the nutrient competition between cancer cells as well as their competition with macrophages during cancer development.^1^ Their data demonstrated that tumor-associated macrophage (TAM) can be dietarily or genetically reprogrammed to win in the competition with MYC-overexpressing cancer cells, providing new insights into our understanding of tumor development and immunotherapy.

Nutrition competition between cells is a phenomenon that commonly exists within organisms and has significant impacts on tissue homeostasis, biological development, and disease progression.^2^ The transcription factor MYC has the ability to confer a “winner” state to cells in the competition during development^3^ and cancer malignancy.^4^ MYC is frequently amplified in malignant cancers, conferring them a super-competitor status in a nutrient competition. However, how the winner state of MYC^hi^ cancer cells is regulated by the intrinsic oncogenic pathways or extrinsic host factors remains poorly understood. Zhang et al. used an autochthonous breast cancer model generated by overexpressing polyomavirus middle tumor antigen (PyMT) with or without MYC overexpression (MYC-PyMT vs. Control-PyMT) to study how the winner state of MYC^hi^ cells being regulated.^1^ They found that MYC accelerated the malignant tumor phenotype, featured by high numbers of large MYC^hi^ cancer cells closely adjacent to the apoptotic MYC^lo^ cancer cells, indicating the existence of cancer cell competition. Furthermore, the authors found the MYC^hi^ cells exhibited active metabolism associated with hyperactivated mTORC1. Intriguingly, when deleting Raptor—the mTORC1 scaffold protein, the MYC^hi^ cancer cells, rather than the MYC^low^ cells, were more prone to die, thereby attenuating the growth of the tumor. These findings indicated the crucial role of mTORC1 signaling in granting competitive fitness to MYC^hi^ cancer cells.

**Fig. 1:**
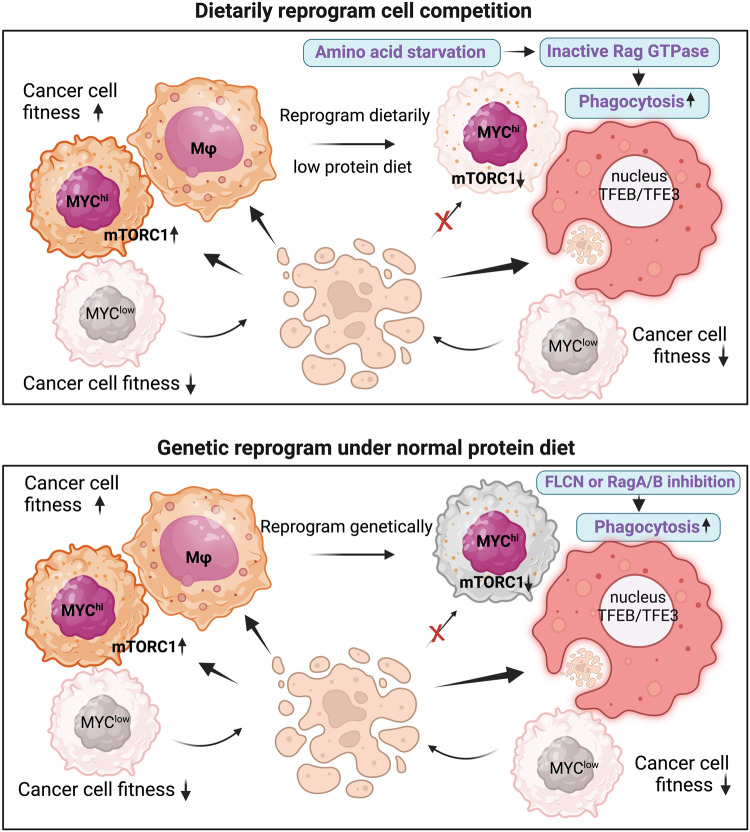
Program TAM to surpass competition from cancer cells. MYC^hi^ cancer cells surpass MYC^lo^ cancer cells in terms of competition fitness that highly relies on mTORC1 signaling. Perturbance of mTORC1 signaling either by a low protein diet abolishes the competitive advantage of MYC^hi^ cancer cells but not that of TAM. Instead, administrating a low protein diet or depleting FLCN (or RagA/B GTPases) of TAM under the normal diet condition enhances the competing fitness of TAM via triggering engulfment of cancer cells mediated by the nuclear translocation and transcriptional activation of TFEB/TFE3. The figure was created with BioRender.com

To further examine how the mTORC1 signaling and transcription factor MYC are coordinated for cellular competition, researchers compared the transcriptome of MYC-PyMT and Control-PyMT tumors. They found MYC-PyMT tumors had higher levels of transcripts responsible for amino acid metabolism and aminoacyl tRNA biosynthesis. Considering the crucial role of mTORC1 signal in nutrients-regulated metabolism, they explored whether the dietary changes could affect MYC/mTORC1-mediated cell competition. Low protein (LP) diet weakened the mTORC1 signaling in MYC-PyMT cancer cells, reversing the competitive fitness between MYC-PyMT and Control-PyMT tumors. Unexpectedly, LP diet significantly enhanced mTORC1 signaling in TAM. Further single-nucleus-RNA-sequencing revealed that TAM from LP diet-treated MYC-PyMT tumor had higher transcripts for phagolysosome biogenesis, phagocytosis, and endocytosis due to the nuclear translocation and transcriptional regulation of TFEB/TFE3. To confirm the role of TFEB/TFE3 in macrophage engulfment, they performed myeloid-specific knockout of TFEB/TFE3 or RPTOR with *Fcgr1-Cre* mouse, which led to suppressed mTORC1 signals of TAMs but restored mTORC1 and growth signals of cancer cells (Fig. 1). These findings demonstrate that LP diet reduces tumor growth by reprogramming TAM into active phagocytic cells in a TFEB/TFE3-dependent manner.

The nuclear translocation of TFEB/TFE3 is controlled by the Rag family of small GTPase whose activity is regulated by GATOR1 and FLCN via sensing cytoplasmic amino acids. When cytoplasmic amino acids were high enough to activate FLCN, the active GTP-bound state of RagA/B and GDP-bound state of RagC/D (RagA/B^GTP^-RagC/D^GDP^) sequesters TFEB/TFE3 in the cytosol. Conversely, when GATOR1 is activated under amino acid starvation, the inactive state of RagA/B^GDP^-RagC/D^GTP^ leads to the dissociation and nuclear translocation of TFEB/TFE3. Therefore, when cytosolic amino acids sensed by the Rag GTPase are reduced under LP diet, the TFEB/TFE3 in TAMs translocate into nuclear for mTORC1 signals and cellular engulfment. Besides, myeloid-specific knockout of Depdc5 in the GATOR1 complex abolished the LP-mediated reprogramming of TAMs by preventing nuclear translocation of TFEB/TFE3. To examine whether the TFEB/TFE3-mTORC1 signaling is sufficient for TAM reprogramming to inhibit MYC-PyMT tumor growth, researchers further knockout FLCN or Rag GTPase in TAM by generating *Fcgr1-cre-Flcn*^*fl/fl*^ and *Fcgr1-cre-Rraga*^*fl/fl*^*Rragb*^*fl/fl*^ mice, and found the enhanced mTORC1 signaling of TAM inhibit tumor growth even under normal diet conditions. These findings suggest the key regulating role of Rag GTPase in TFEB/TFE3 nuclear translocation and mTORC1 signaling, targeting which can reprogram TAM for tumor suppression.

Notably, apoptotic cell engulfment of macrophages is the key to their competitive superiority over cancer cells in nutrient competition. They found that the TAMs from LP diet-treated tumor exhibited higher apoptotic cell-engulfment activity, whereas the cancer cells showed increased apoptosis due to the lack of mTORC1 signaling for competitive fitness. To further demonstrate the importance of engulfment in TAMs reprogramming, researchers knocked out the lipid kinase PIKfyve in macrophages, which resulted in impaired engulfment activities of TAMs but the restored mTORC1 signaling and growth of cancer cells. Knockout of PIKfyve in cancer cells led to weakening mTORC1 and retarded tumor growth regardless of the TAMs phenotype. Collectively, these results demonstrate that mTORC1 is a key fitness determinant for both macrophage and cancer cells, and the engulfment-mediated nutrient acquisition grants TAM a unique advantage over cancer cells, which might be a promising way to promote anti-tumor immunity.

Cell competition plays a critical role in determining the outcome of the disease. mTOR is a serine/threonine kinase that integrates various environmental signals and nutritional status to direct and optimize cell growth and inflammatory responses. In particular, mTOR signaling has been shown as a key coordinator in macrophage polarization. For example, mTORC2 signaling is required for M2 macrophage polarization, whereas mTORC1 signaling contributes to proinflammatory M1 rather than M2 polarization.^5^ Notably, several mTOR inhibitors, such as Rapamycin and Sirolimus, have been approved for cancer treatment, but the overall clinical efficacy is restricted, which might be due to their unscrutinized effects on immune cells such as macrophages and T cells. Therefore, precisely manipulating the mTORC1 or mTORC2 signals rather than simply blocking mTOR signaling might be a promising direction in leveraging macrophage for anti-tumor immunity. This study demonstrated that dietary control can modulate macrophage polarization and the balance of cell competition to enhance cancer therapy. By scrutinizing the competition between macrophages and cancer cells, this study has demonstrated a novel dietary pathway for cancer inhibition. The shift from cooperation to competition provides a new path to design combinatorial immunotherapy for malignant cancers.
